# Is matching or discrepancy between filial piety expectation and filial support better? The role of filial support of children and social support

**DOI:** 10.1002/pchj.752

**Published:** 2024-04-15

**Authors:** Xiying Li, Xingyu Zhang, Jing Liu, Yuxiang Cao, Zhongling Pi, Huamao Peng

**Affiliations:** ^1^ MOE Key Laboratory of Modern Teaching Technology Shaanxi Normal University Xi'an China; ^2^ Key Research Base of Humanities and Social Sciences of the Ministry of Education, Academy of Psychology and Behavior Tianjin Normal University Tianjin China; ^3^ Department of Educational and Psychological Science Yuncheng University Yuncheng China; ^4^ School of Educational Science Anhui Normal University Anhui China; ^5^ Institute of Developmental Psychology Beijing Normal University Beijing China

**Keywords:** filial responsibility expectation, filial support, life satisfaction, loneliness, social support

## Abstract

Filial piety in traditional Chinese culture is an essential variable in explaining intergenerational interaction. However, previous studies have not clarified whether older adults' filial responsibility expectations matched children's filial support and the effects of the filial discrepancy on their life satisfaction and loneliness. The latent profile analysis showed that older adults were divided into two groups: (1) high expectations and support, and (2) low expectations and support. The results showed that compared with older adults with low expectations and low support, those with high expectations and high support reported higher life satisfaction and lower loneliness. Additionally, social support played a moderating role in the effect of the groups of older adults on life satisfaction and loneliness. Our conclusion shows that filial support is an essential factor influencing older adult life satisfaction and loneliness, and social support is an effective supplement to filial support.

## INTRODUCTION

“Of all virtues, filial piety is the most important,” is rooted in traditional Chinese culture, and it is an important variable to explain the intergenerational interaction in the context of Chinese culture (Wang et al., 2009). Filial responsibility expectation is a social attitude and belief in filial piety, in which older adults think their adult children have responsibilities to meet their needs, not only reflects the general social norms, but also focuses on the specific behaviors toward their children (van der Pas et al., 2005).

The traditional belief in filial piety makes older adults always have high filial responsibility expectations for their children (Ren et al., 2022). However, with the rapid development of the economy and the evolution of human civilization, older adults' filial responsibility expectation is changing (Cheng & Chan, 2006; Chong & Liu, 2016; Hsu et al., 2001; Seelbach, 1977; van der Pas et al., 2005). For example, older adults satisfy most of their instrumental needs themselves (Cheng & Chan, 2006). Therefore, the contents and degree of older adults' filial responsibility expectations are changing (Fu et al., 2016; Guo et al., 2020; Ren et al., 2022). Some researchers claim that as older adults' filial responsibility expectations change, adult children regulate their behaviors to match the expectations to reduce filial discrepancy (Bai, 2019; Ren et al., 2022). Some emerging studies have shown that adult children's support matches older adults' filial responsibility expectations of financial, material, and information care (Bai, 2019). Additionally, the higher the filial responsibility expectation of older adults, the higher the actual filial support because the children perceive their high expectations (Wang et al., 2013).

Furthermore, adult children's behaviors related to filial responsibility are also changing. As urbanization increases, the size of families decreases, the geographical distance increases, and adult children are increasingly unable to directly care for their elderly parents at home due to work and other related responsibilities. To avoid disappointment and burden on their children, older adults tend to suppress their expectations and find other ways to meet their needs (Ren et al., 2022). For example, older adults seek support from the government, friends, and neighbors (van der Pas et al., 2006). Therefore, they will reduce filial discrepancy and gain a new balance.

Taken together, filial responsibility expectation is adaptive, especially in China. China is a relationship‐oriented culture and Chinese commonly believed that disharmonious family relationships might lead to a decline in overall life satisfaction (Huang & Fiocco, 2020). Consequently, older adults' filial responsibility expectations and adult children's behaviors interactively regulate to match each other.

There is considerable interest in the relationship between filial responsibility expectation and older adults' quality of life (Li & Dong, 2018; Zheng & Li, 2022). However, previous studies did not produce consistent conclusions (Lee et al., 1995; Luo et al., 2019; Wang et al., 2019). For example, some studies showed that the filial responsibility expectation was positively correlated with subjective well‐being (Chen & Fang, 2013; Wang et al., 2009; Wang et al., 2019) and was negatively associated with loneliness and depression in older adults (Li & Dong, 2018; Luo et al., 2019). In contrast, Seelbach and Sauer (1977) pointed out that the higher expectations of child care during parental illness, the lower the life satisfaction. Lee et al. (1995) also observed a similar phenomenon, stating that high filial responsibility expectation was associated with higher rates of depression.

The mixed results might result from not considering filial support behaviors from their adult children and filial discrepancy (Dong et al., 2017; Liu et al., 2020). Filial responsibility expectation is the expectation of parents to receive filial support, and filial support behavior is the actual supportive behavior of parent–child interaction (Chen & Fang, 2013). Some researchers argued that there might be a filial discrepancy, and the discrepancy might affect their satisfaction, subjective well‐being, and mental health (Dong et al., 2012; Hamon & Blieszner, 1990; Liu et al., 2020; Zheng & Li, 2022). For example, Liu et al. (2020) found that filial discrepancy and two emotional‐support domain discrepancies (respect and greeting) were associated with depressive symptoms and stress.

Filial support behavior is a kind of intergenerational social support from older adults' children. For older adults, the family is the primary provider of social support, especially regarding material/financial support and activities of daily living (Dai, 1995). To have a high quality of life, they first seek social support from their children. If they cannot get social support from their children, they seek social support outside the family, such as from friends and neighbors (Dong & Zhang, 2016a). For example, a study by Dong and Zhang (2016b) found that many older adults receiving a public pension from American social welfare decreased their filial responsibility expectations because their needs were met. Additionally, when the social support that older adults receive outside the family increases, they would reduce the need for children's filial support. Therefore, social supports outside the family compensate for the shortage of social support within the family. It is reasonable to assume that social support outside the family moderates the effects of the filial discrepancy on their quality of life.

However, few studies have considered whether older adults' filial responsibility expectations match children's filial support and the effects of the filial discrepancy on their life satisfaction and loneliness. Despite the importance of social support outside the family for older adults, previous studies have paid little attention to the moderation role of such kinds of social support to their quality of life. To bridge the gap, the present study had three research questions.

Do older adults' filial responsibility expectations match children's filial support behaviors by latent profile analysis to classify older adults?

Does filial discrepancy affect older adults' life satisfaction and loneliness?

Does social support outside the family moderate the effect of the filial discrepancy on life satisfaction and loneliness of older adults?

Therefore, we propose the following research hypotheses:

Hypothesis 1 According to the relationship between expectation and support, there are four types: high expectation with high support (HH), low expectation with low support (LL), high expectation with low support (HL) and low expectation with high support (LH), where HH or LL indicate low discrepancy and HL or LH indicate high discrepancy.

Hypothesis 2 Compared with HL and LH, HH and LL have higher life satisfaction and lower loneliness. Compared with LL, HH had higher life satisfaction and lower loneliness.

Hypothesis 3 Social support outside the family could moderate the effect of the filial discrepancy on life satisfaction and loneliness of older adults.

## METHODS

### Study design and participants

The present study was a cross‐sectional survey conducted in different socioeconomic areas of Chongqing, a city in Southwestern China. The protocol was approved by the Ethical Committee of Shaanxi Normal University. All participants signed written informed consent. We recruited 201 participants from different socioeconomic areas of Chongqing through convenience sampling, with 91 men (39.4%), 109 women (60.0%), and one participant who declined to identify their sex. The age ranges between 59 and 77 years, with a mean age of 68.05 years (*SD* = 9.04); four participants did not indicate their age. As to educational level, 32 participants had a primary school degree or below (15.9%), 53 had a junior high school degree (26.4%), 58 had senior high school or technical school (28.9%), 35 had college degrees (17.4%), 20 had bachelor degrees or above (10.0%), and three did not provide the information (Table 1).

**TABLE 1:** The distribution of participants.

	*N*	Percentage (%)
Gender		
Male	91	39.40
Female	109	60.00
Missing	1	0.50
Marital status		
Unmarried	23	11.40
Married	174	86.60
Missing	4	2.00
Degree of education		
Primary school and below	32	15.90
Junior high school	53	26.40
High school/Technical school	58	28.90
College	35	17.40
Bachelor degree or above	20	10.00
Missing	3	1.50
Cohabitant		
Spouse	110	53.70
Children	13	6.50
Spouse and children	52	25.90
Living alone	23	11.40
Others	2	1.00
Missing	1	0.50

### Measures

#### Filial responsibility expectation

We used the Chinese Filial Responsibility Scale (CFRS) to measure the filial responsibility expectation of older adults (Li et al., 2023; van der Pas et al., 2005). It contains 16 items with four dimensions: emotional expectation (five items), instrumental expectation (five items), contact expectation (three items), and information expectation (three items). Participants rated each item on a 5‐point scale (1 = *strongly disagree*, 5 = *strongly agree*). Higher scores indicate higher levels of filial expectations. In this study, the Cronbach's alpha reliability coefficient of the CFRS was .96. The Cronbach's alpha reliability coefficient of emotional expectation, instrumental expectation, contact expectation, and information expectation were .90, .91, .82, and .86, respectively.

#### Parents‐adult children social support

We modified the Parents‐Adult Children Social Support Scale (PACSS) to measure the children's filial support (Shen et al., 2003). The revised PACSS contains 20 items and four dimensions, including emotional support, tool support, contact support, and information support. According to the frequency of social support from children, older adults answered each item from 1 (*never*) to 4 (*always*). In this study, the Cronbach's alpha reliability coefficient of emotional support, instrumental support, contact support, information support, and the total score were .92, .89, .78, .86, and 0.96, respectively.

#### Life satisfaction

We used the Satisfaction With Life Scale (SWLS) to measure life satisfaction, consisting of five items (Diener et al., 1985). Participants responded to the items on a 7‐point scale ranging from 1 = *strongly disapprove*’ to 7 = *strongly approve*. The higher the score, the higher the life satisfaction. In this study, the Cronbach's alpha reliability coefficient was .97.

#### Social support

We used the Chinese version of the Social Support Questionnaire for Transactions to measure social support outside the family (SSQT; Sit et al., 2004; Suurmeijer et al., 1995). This questionnaire included 23 items in five dimensions: daily emotional support (DES), problem‐oriented emotional support (PES), social companionship (SC), daily instrumental support (DIS), and problem‐oriented instrumental support (PIS). Participants responded to each item on a scale ranging from 1 = *seldom or never* to 4 = *often*. Higher scores indicate higher levels of social support. In this study, the Cronbach's alpha reliability coefficient for the five dimensions and the total score were .85, .89, .90, .88, .80, and .96, respectively.

#### Loneliness

We used the Revised UCLA Loneliness Scale (R‐UCLA‐LS) to measure loneliness (Ausín et al., 2018). This scale included 20 items. Participants responded to each item on a scale ranging from 1 = *strongly disagree* to 4 = *strongly agree*, and the overall score ranged from 20 to 80 points. Higher scores indicate a higher level of perceived loneliness. In the present study, the Cronbach's alpha reliability coefficient was .80.

### Data analytical strategy

We used the latent profile analysis (Mplus 8.0) to classify older adults according to scores of filial responsibility expectation and children's filial support behavior. Latent profile analysis is a clustering method that aims to classify individuals into different groups based on their response patterns to multiple variables. We established 3–4 models at different grouping levels and then compared the goodness‐of‐fit using the Akaike Information Criterion (AIC), Bayesian Information Criterion (BIC), adjusted BIC (aBIC), and so on, to determine the optimal model that would provide the most suitable grouping level for the current dataset. Then we used SPSS28.0 to examine the differences in life satisfaction and loneliness between the various groups of older adults. Finally, we used Process to examine the moderating effect of social support on these differences.

## RESULTS

### Descriptive statistics and correlation analysis

Table 2 shows descriptive statistics for all variables and the correlation results, exhibiting the most significant correlations among variables (*p*s < .01).

**TABLE 2:** Descriptive statistics and correlations.

	*M* ± *SD*	1	2	3	4	5	6	7	8	9	10	11	12	13	14	15	16	17
1. EE	4.369 ± 0.580	1																
2.ISE	3.355 ± 0.668	0.682**	1															
3.CE	4.355 ± 0.668	0.708**	0.588**	1														
4. IFE	4.244 ± 0.708	0.746**	0.630**	0.666**	1													
5. FE	16.549 ± 2.484	0.888**	0.865**	0.841**	870**	1												
6. ES	3.180 ± 0.628	0.421**	0.340**	0.350**	0.370**	0.423*	1											
7.ISS	3.347 ± 0.597	0.420**	0.338**	0.348**	0.426**	0.438**	0.710**	1										
8. CS	3.460 ± 0.585	0.420**	0.284**	0.408**	0.415**	0.431**	0.770**	0.740**	1									
9. IFS	3.484 ± 0.560	0.405**	0.226**	0.331**	0.372**	0.373**	0.771**	0.781**	0.78**	1								
11. FS	13.407 ± 2.143	0.461**	0.330**	0.397**	0.437**	0.461**	0.902**	0.892**	0.908**	0.917**	1							
10. LS	26.362 ± 5.450	0.252**	0.175**	0.254**	0.293**	0.275**	0.207**	0.261**	0.33**	0.252**	0.289**	1						
12. LN	36.379 ± 8.659	−0.09	0.038	−0.015	−.164*	−.058	−0.103	−0.099	−0.176*	−0.117	−0.424**	−0.099	1					
13. SS	69.635 ± 13.434	−0.048	0.035	0.012	0.093	.356**	−0.075	0.059	−0.021	0.007	−0.022	0.284**	0.158*	1				
14. DES	16.080 ± 2.885	0.299**	0.314**	0.282**	0.269**	0.335**	0.472**	0.300**	0.412**	0.317**	0.337**	0.299**	−0.242**	−0.069**	1			
15. PES	18.935 ± 3.567	0.256**	0.273**	3.10E+03	0.278**	0.324**	0.489**	0.309**	0.350**	0.328**	0.247**	0.309**	−0.156*	−0.046	0.723**	1		
16. SC	15.238 ± 3.784	0.171*	0.194*	0.210**	0.189*	0.222**	0.412**	0.248**	0.371**	0.277**	0.289**	0.247**	−0.263**	−0.158**	0.672**	0.701**	1	
17. DIS	7.274 ± 2.784	0.272**	0.318**	0.239**	0.267**	0.321**	0.530**	0.390**	0.349**	0.388**	0.145*	0.403**	0.053	−0.034	0.499**	0.648**	0.559**	1
18. PIS	12.100 ± 2.748	0.291**	0.223**	0.339**	0.285**	0.311**	0.427**	0.413**	0.386**	0.355**	0.347**	0.382**	−0.245**	−0.065**	0.568**	0.636**	0.620**	0.651**

Abbreviations: CE, contact expectation; CS, contact support; DES, daily emotional support; DIS, daily instrumental support; EE, emotion expectation; ES, emotion support; FE, total score of filial expectation; FS, total score of filial support; IFE, information expectation; IFS, information support; ISE = instrumental expectation; ISS, instrumental support; LN, loneliness; LS, life satisfaction; PES, problem‐oriented Emotional support; PIS, problem‐oriented instrumental support; SC, social companionship.

* *p* < .05, ** *p* < .01, *** *p* < .001.

### Latent profile analysis

To answer our first research question of whether older adults' filial responsibility expectations matched children's filial support behaviors, we used latent profile analysis to categorize the older adults according to four dimensions of filial responsibility expectation (emotional expectation, instrumental expectation, contact expectation, information expectation) and four dimensions of filial support behavior (emotional support, instrumental support, contact support, information support). Table 3 shows that AIC, BIC, and aBIC gradually decreased from Model 1 to Model 4, but the Lo–Mendell–Rubin likelihood ratio tests (LMR) of Models 3 and 4 were no longer significant. Therefore, Model 2, which divided older adults into two groups, was relatively best compared to the other models.

**TABLE 3:** Comparison of indicators of model fit for latent profile analysis.

Model	Log (L)	AIC	BIC	aBIC	Entropy	LMR	BLRT	Probability of categories
1	−1577.77	3187.533	3240.386	3189.695				
2	−1274.99	2599.974	2682.557	2603.353	0.907	593.13*	−1577.77*	0.318/0.682
3	−1148.06	2364.123	2476.435	2368.718	0.906	248.64	−1274.99**	0.343/0.199/0.458
4	−1073.89	2233.776	2375.818	2239.588	0.904	145.30	−1148.06**	0.258/0.468/0.059/0.214

Abbreviations: aBIC, adjusted BIC; AIC, Akaike Information Criterion; BIC, Bayesian Information Criterion; BLRT, bootstrapped likelihood ratio test; L, loglikelihood; LMR, Lo–Mendell–Rubin.

* *p* < .05, ** *p* < .01, *** *p* < .001.

Table 4 shows the probabilities of the two categories. The average probabilities of the older adults in each category were 97.7% for Group 1 (G1) and 98.3% for Group 2 (G2), suggesting that classifying the older adults into two groups was suitable and reliable.

**TABLE 4:** Average probability of each potential group.

	G1 (%)	G2 (%)
G1	0.964	0.036
G2	0.026	0.974

**FIGURE 1:**
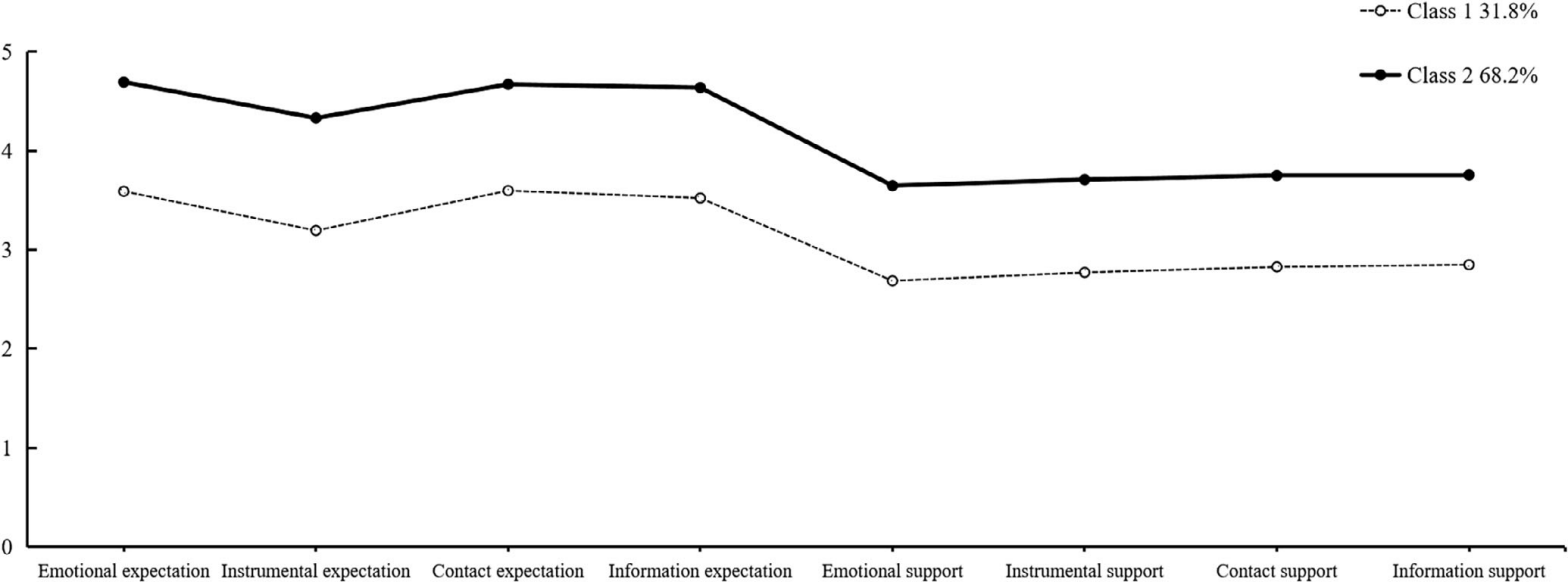
Response probability of HH and LL.

We further obtained the response probability graph of G1 and G2 in eight dimensions of filial responsibility expectation and filial support. As Figure 1 shows, G2 scored higher than G1 in all dimensions. Therefore, we defined G2 as a high expectation and support group (HH), which accounted for 68.2% of all participants; and G1 was defined as a low expectation and support group (LL), accounting for 31.8%. The above results suggested that older adults' filial responsibility expectations matched children's filial support, regardless of their filial responsibility expectations.

### The differences in life satisfaction and loneliness between HH and LL

To answer our second research question of whether different groups of older adults influenced older adults' life satisfaction and loneliness, we conducted the independent sample *t*‐tests showing that the life satisfaction in HH was significantly greater than in LL (*MD* = 3.21, *t* (1,99) = 4.03, *p* < .001), and the loneliness in HH was lower than LL at a marginally significant level (*MD* = −2.28, *t* (1,99) = −1.75, *p* = .081). Our results showed that older adults with low expectations and support reported greater life satisfaction and heavier loneliness than older adults with high expectations and support.

### The moderating effects of social support outside family

**FIGURE 2:**
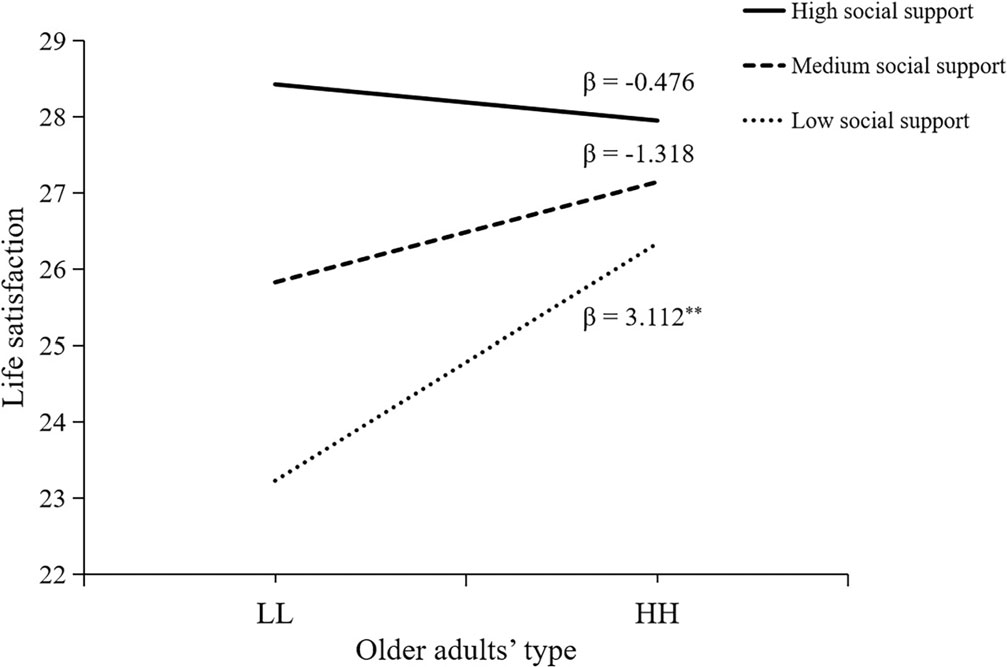
The moderating effect of social support for life satisfaction. HH = High expectation and support group; LL = Low expectation and support group.

We analyzed the moderating effects of social support to answer our third research question. In terms of life satisfaction, social support had a significant moderating effect on the relationship between older adults' groups and life satisfaction (*∆R* ^2^ = 0.019, *F*(1,197) = 4.43, *p* = .037; Table 5). A further simple slope test (see Figure 2) did not show significant differences in life satisfaction between the LL and HH groups when social support was high and medium. However, when social support was low, life satisfaction in the HH group was significantly higher than in the LL group (β = 3.11, *t* = 3.04, *p* = .003). Therefore, our results suggest that social support was a beneficial supplement to children's support for life satisfaction of older adults and that high and medium social support could compensate for the negative effect of low children's support on the life satisfaction of older adults.

**TABLE 5:** Moderation effects of social support on the difference in life satisfaction.

	Coefficient	SE	*p*	∆*R* ^2^	*F*	*p*
Intercept	25.826	0.775	<.001	.019	4.425	.037
Type	1.318	0.900	.145			
Social Support	2.600	0.703	<.001			
Type * Social Support	−1.794	0.853	.037			
Intercept	25.266	0.709	<.001	.001	1.862	.174
Type	1.858	0.837	.028			
DES	2.233	0.655	<.001			
Type * DES	−1.097	0.809	.174			
Intercept	25.146	0.753	<.001	.010	2.204	.139
Type	−2.100	0.881	.018			
PES	1.847	0.733	.013			
Type * PES	−1.288	0.878	.139			
Intercept	25.071	0.695	<.001	.011	2.600	.108
Type	2.159	0.827	.010			
SC	1.972	0.599	.001			
Type * SC	−1.251	0.776	.108			
Intercept	25.295	0.841	<.001	.020	4.356	.038
Type	2.145	0.960	.027			
DIS	1.888	0.896	.036			
Type * DIS	−2.094	1.004	.038			
Intercept	25.632	0.773	.001	.007	1.659	.199
Type	1.401	0.899	.121			
PIS	2.219	0.671	.001			
Type * PIS	−1.080	0.838	.199			

*Note*: Type: 0 = low expectation and low support group; 1 = high expectation and high support group.

Abbreviations: DES, daily emotional support; DIS, daily instrumental support; PES, problem‐oriented emotional support; PIS, problem‐oriented instrumental support; SC, social companionship.

**FIGURE 3:**
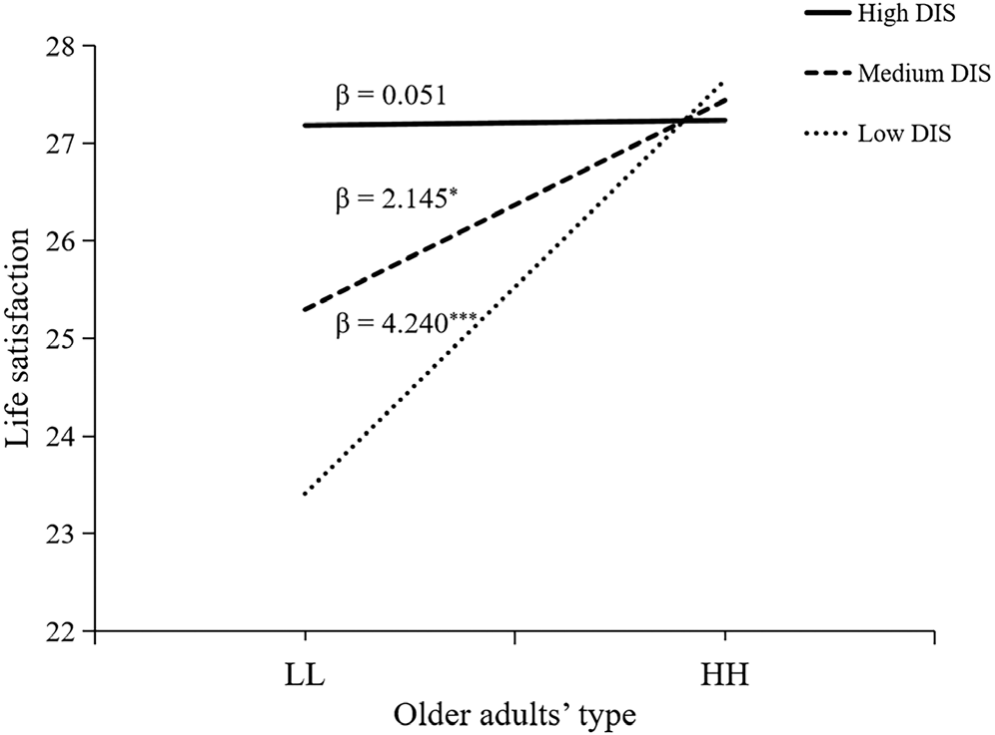
The moderating effect of daily instrumental support (DIS) for life satisfaction. HH = High expectation and support group; LL = Low expectation and support group.

Then, we tested moderating effects of five dimensions of social support (Table 5). The results did not show any significantly moderating effect of DES (*∆R* ^2^ = 0.001, *F*(1,197) = 1.86, *p* = .174), PES (*∆R* ^2^ = 0.010, *F*(1,197) = 2.20, *p* = .139), SC (*∆R* ^2^ = 0.011, *F*(1,197) = 2.60, *p* = .108), and PIS (*∆R* ^2^ = 0.007, *F*(1,197) = 1.66, *p* = .199), whereas the moderating effect of DIS was significant (*∆R* ^2^ = 0.020, *F*(1,197) = 4.37, *p* = .038). A further simple slope test (see Figure 3) did not show significant differences in life satisfaction between the LL and the HH groups when DIS was high. However, when DIS was medium, life satisfaction in the HH group was significantly higher than that in the LL group (β = 2.15, *t* = 2.24, *p* = .027); and when DIS was low, life satisfaction in the HH group was also significantly higher than that in the LL group (β = 4.24, *t =* 4.06, *p <* .001). Therefore, our results show that, in terms of the life satisfaction of older adults, the supplementary role of social support is mainly reflected in the daily instrumental support of social support. The high level of daily instrumental support can make up for the effect of children's low support on their life satisfaction.

**FIGURE 4:**
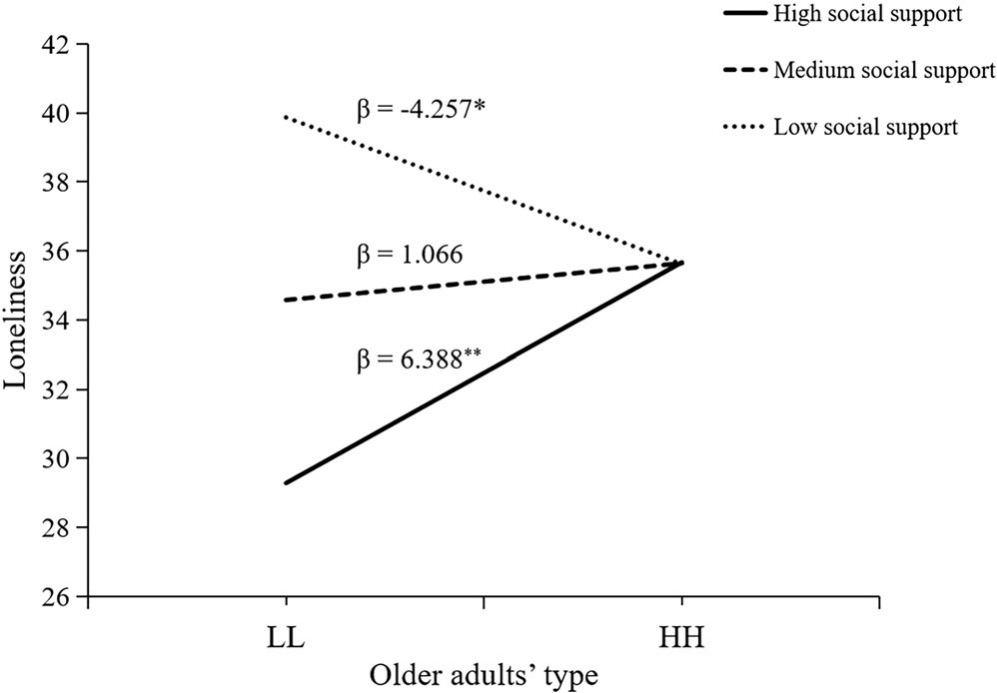
The moderating effect of social support for loneliness. HH = High expectation and support group; LL = Low expectation and support group.

We tested moderating effects of social support for loneliness. The results in Table 6 showed that there were significant moderating effects of social support (*∆R* ^2^ = 0.067, F (1,197) = 14.82, *p* < .001). We conducted further simple slope tests (Figure 4). When social support was low, loneliness in the HH group was significantly higher than that in the LL group (β = 6.39, *t* = 6.22, *p* = .005); when social support was low, loneliness in the HH group was significantly lower than that in the LL group (β = −4.26, *t* = −4.14, *p* = .03).

**TABLE 6:** Moderating effects of social support on the difference in loneliness.

	Coefficient	S.E.	*p*	∆*R* ^2^	*F*	*p*
Intercept	34.577	1.256	<.001	.067	14.815	<.001
Type	1.066	1.458	.466			
Social Support	−5.294	1.140	<.001			
Type * Social Support	5.323	1.383	<.001			
Intercept	35.474	1.148	<.001	.057	12.735	<.001
Type	0.269	1.356	.843			
DES	−5.046	1.062	<.001			
Type * DES	4.648	1.302	<.001			
Intercept	35.882	1.226	<.001	.037	7.741	.006
Type	−0.236	1.434	.870			
PES	−3.911	1.193	.001			
Type * PES	3.931	1.413	.006			
Intercept	35.908	1.115	<.001	.044	9.709	.002
Type	−0.132	1.328	.921			
SC	−4.469	0.962	<.001			
Type * SC	3.880	1.245	.002			
Intercept	36.586	1.363	<.001	.032	6.667	.011
Type	−1.466	1.556	.347			
DIS	−2.278	1.452	.118			
Type * DIS	4.201	1.626	.011			
Intercept	35.338	1.272	<.001	.214	4.601	.033
Type	0.619	1.481	.676			
PIS	−3.960	1.106	<.001			
Type * PIS	2.961	1.381	.033			

Abbreviations: DES, daily emotional support; DIS, daily instrumental support; PES, problem‐oriented emotional support; PIS, problem‐oriented instrumental support; SC, social companionship.

We also tested moderating effects of five dimensions of social support. The results in Table 6 showed that there were significant moderating effects of DES (*∆R* ^2^ = 0.057, *F*(1,197) = 12.74, *p* < .001), PES (*∆R* ^2^ = 0.037, *F*(1,197) = 7.74, *p* = .006), SC (*∆R* ^2^ = 0.044, *F*(1,197) = 9.71, *p* = .002), DIS (*∆R* ^2^ = 0.032, *F*(1,197) = 6.67, *p* = .011), and PIS (*∆R* ^2^ = 0.021, *F*(1,197) = 4.60, *p* = .033). We conducted further simple slope tests (Figure 4). Regarding DES, when DES was low, loneliness in the HH group was significantly lower than that in the LL group (β = −4.38, *t* = −2.67, *p* = .008); when DES was medium, there were no significant differences in loneliness between HH and LL groups; when DES was high, loneliness in HH group was significantly higher than that in LL group (β = 4.92, *t* = 2.09, *p* = .020).

**FIGURE 5:**
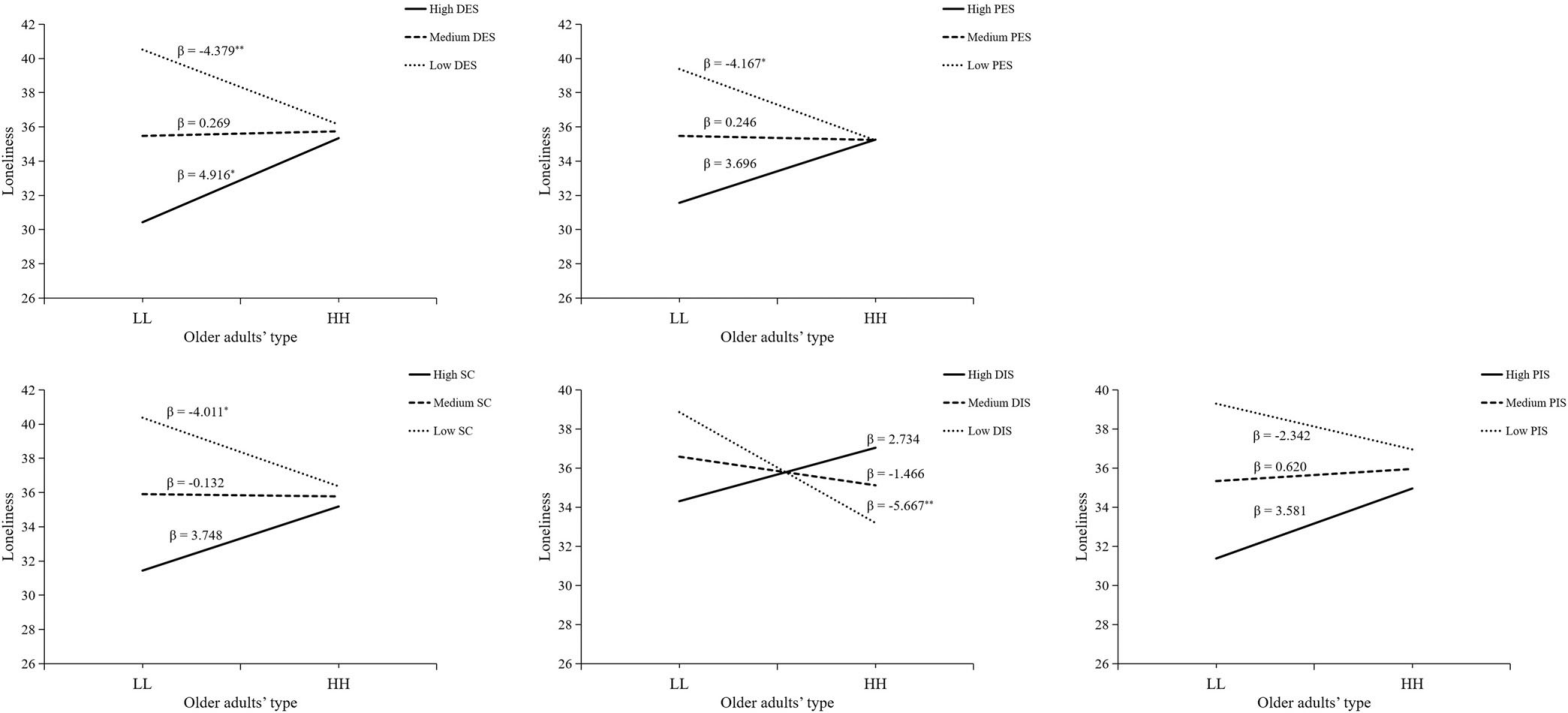
The moderating effect of five dimensions of social support for loneliness. HH = High expectation and support group; LL = Low expectation and support group.

Results of PES, SC, and DIS were similar (Figure 5). When PES, SC, and DIS were low, loneliness in the HH group was significantly lower than that in the LL group (β = −4.17, *t* = −2.47, *p* = .014), but there were no differences in loneliness between the HH and LL groups when PES, SC, and DIS was medium and high. Therefore, our results suggest that medium DES or medium or high PES, SC, and DIS can compensate for the negative effect of low child support on loneliness in older adults. However, high DES increases the loneliness of older adults with high expectations and support.

## DISCUSSION

The present study aimed to test the filial discrepancy between older adults' filial responsibility expectations and children's filial support, and the moderating effects of social supports outside the family on older adults' life satisfaction and loneliness. The results of our study show that older adults' filial responsibility expectations matched their children's filial support. Compared with older adults with low expectations and support, those with high expectations and support had higher life satisfaction and lower loneliness. Furthermore, social support played a moderating role in the effect of the filial discrepancy on life satisfaction and loneliness.

Regarding our first research question, the results of latent profile analysis recognized two types of older adults: one was a high expectation and support group, and the other was a low expectation and support group. This result did not validate our hypothesis. This may be due to the shortcomings of our research methods. Our measures of filial expectations and child support are subjective reports from older adults, rather than objective measures, which we also address in the limitations of our study. Some previous studies also suggested a positive tendency to evaluate their children's filial support and the behavioral confirmation effect during interpersonal interaction (Blanck et al., 1993; Ren et al., 2022). On the other hand, despite the development of the economy and integration of Chinese and Western cultures, Chinese older adults still have a high filial responsibility expectation (Li et al., 2023; Ren et al., 2022). Elderly parents often justify their children's failure to comply with expectations. For example, they explain that their children have their own lives and families and are busy with work. Consequently, they tend to evaluate their children's support positively (Ren et al., 2022). In other words, they think that their children provide high support. On the other hand, people expect that the desired object may behave the same way as the perceiver, which is called the behavioral confirmation effect (Blanck et al., 1993). The perceiver may initiate some verbal or non‐verbal behaviors to convey their expectation, thus implying the desired behavior. If the behavioral confirmation does not occur accordingly, perceivers may change their expectations after a period of observation (Blanck et al., 1993; Dai, 1995). Therefore, when there is a filial discrepancy, they restrain their expectations and gradually match their children's low filial support to avoid disappointment.

Regarding our second research question, we found that such restraining reduced older adults' life satisfaction and increased loneliness. The results were consistent with previous studies on older adults (Dai, 1995; Li et al., 2019). According to the social convoy model, family is the core of a person's social relations (Li et al., 2017), and Dai (1995) also believe that the life of older adults is not centered around themselves, but around their family (Li et al., 2019). Children's filial support is crucial to improving older adults' life satisfaction and reducing loneliness. Although older adults would regulate their expectations to match their children when children provide low filial support, they hope to get high filial support. High expectations and high support mean a harmonious family relationship, because the expression and transmission of filial responsibility expectations accelerates the information flow between parents and children, and increases children's filial support. Although older adults with low expectations and support also achieved a balance, this balance was a situation in which older adults suppressed their expectations to avoid disappointment and burdening their children. In essence, it was a compromise caused by the low support. Therefore, older adults with low expectations and support had less life satisfaction and greater loneliness than those with high expectations and support.

Regarding our third research question, we observed the moderating role of social support outside the family, which supported the complementarity hypothesis (Künemund & Rein, 1999). According to this hypothesis, social support supplements family support (Künemund & Rein, 1999; Motel‐Klingebiel et al., 2005). Our results suggested that social support was a beneficial supplement to children's support for life satisfaction of older adults. Specifically, high social support (mainly reflected in the daily instrumental support of social support) compensated for the negative effect of children's low support on older adult’ life satisfaction. However, when social support (mainly reflected in the daily instrumental support) was high, the difference in life satisfaction between the HH and LL groups was reduced. The complementarity hypothesis of social support holds that complementarity of social support can be realized through family specialization or task‐specific complementarity (Litwak, 1985; Lyons et al., 2000). Family and social support provide different kinds of support. Specifically, families concentrate on emotional support, and social support focuses on instrumental support (Litwak, 1985; Lyons et al., 2000). Daily instrumental support can solve the difficulties and problems that older adults with low expectations and support encounter in everyday life, which is conducive to compensating for the negative effect of insufficient family support on the life satisfaction of older adults.

In addition, our results suggested that medium or high social support (except daily emotional support) compensated for the negative effect of children's low support on older adults' loneliness. Surprisingly, high daily emotional support increased the loneliness of older adults with high expectations and support. Silverstein et al. (1996) found that oversupport (low expectations, high support) had a more negative effect on depression than undersupport (high expectations, low support). They argue that receiving support in undesired amounts compromises the independence and subsequently reinforces a decline in well‐being. Therefore, the (im)balance between the desired and actual situation seems fundamental when studying child contact and support and parental loneliness. Some researchers also believe that receiving social support reduces the stability of subjective well‐being in older adults (Sarason et al., 1990). Receiving social support may evoke feelings of incompetence, which are moderated by someone's need for support (Deelstra et al., 2003). Therefore, the activation of social support is a diverse process. Whether you need support, whether you are seeking support or passively accept support, and whether the recipient of support is satisfied, might be critical in determining the effectiveness of received support (Chalise et al., 2007).

There are some limitations that need to be further studied in the future. Firstly, this study used the PACSS reported by older adults rather than their children, which might generate biased or inaccurate results. Older adults' responses may be shaped by social desirability, and they tend to positively evaluate their children's support (Ren et al., 2022). Future studies need to test whether there is filial discrepancy by directly measuring children's support. Secondly, the results of latent profile analysis recognized two types of older adults: one was the consistency of high expectation and support group, and the other was the consistency of low expectation and support group. In the present study, we did not observe filial discrepancy, which existed in other Chinese older adults in the United States (e.g., Chinese immigrant adults; Liu et al., 2020). Therefore, we could not compare the differences among the four groups of older adults and whether there were differences in the supplementary effects of social support in the four types of older adults. The grouping results may be due to a small sample size or bias, or they may reflect the situation in mainland China only. Further studies need to address these questions with a large sample size.

## CONFLICT OF INTEREST STATEMENT

The authors declare there are no conflicts of interest.

## ETHICS STATEMENT

The protocol was approved by the Ethical Committee of the Shaanxi Normal University (Ethical Approval Reference Number: SNNU2021060126).

## Data Availability

Our data and material are not yet available online in any institutional database. However, the whole data package and material are available by request to Professor Zhongling Pi: pizl@snnu.edu.cn.
